# Fighting Strategies Against Chagas’ Disease: A Review

**DOI:** 10.3390/pathogens14020183

**Published:** 2025-02-12

**Authors:** Andrea Hernández-Flores, Debora Elías-Díaz, Bernadeth Cubillo-Cervantes, Carlos N. Ibarra-Cerdeña, David Morán, Audrey Arnal, Andrea Chaves

**Affiliations:** 1 Departamento de Etología, Fauna Silvestre y Animales de Laboratorio, Facultad de Medicina Veterinaria y Zootecnia, Universidad Nacional Autónoma de México (UNAM), Ciudad Universitaria, Av. Universidad #3000, Mexico City 04510, Mexico; andreahf.mvz@gmail.com (A.H.-F.); audrey.arnal@ird.fr (A.A.); 2 Sistema de Estudios de Posgrado Posgrado en Biología, Universidad de Costa Rica, San José 11501-206, Costa Rica; debaelias@gmail.com (D.E.-D.); bernadethcubillo21@gmail.com (B.C.-C.); 3 Departamento de Ecología Humana, Centro de Investigación y de Estudios Avanzados (Cinvestav), Unidad Mérida, Merida 97205, Mexico; ibarra.cerdena@gmail.com; 4 Unidad de Ecología y Epidemiología, Programa Arbovirus y Zoonoses, Centro para Estudios de Salud, Universidad del Valle de Guatemala, Guatemala City 01015, Guatemala; dmoran@uvg.edu.gt; 5 MIVEGEC, IRD, CNRS, Université de Montpellier, 34394 Montpellier, France; 6 International Joint Laboratory IRD/UNAM ELDORADO, Merida 97205, Mexico; 7 Centro Nacional de Innovaciones Biotecnológicas (CENIBiot), CeNAT, Conare, San José 1174-1200, Costa Rica; 8 Escuela de Biología, Universidad de Costa Rica, San José 11501-206, Costa Rica

**Keywords:** *Trypanosoma cruzi*, vector control, surveillance systems, public health strategies, neglected diseases

## Abstract

Chagas disease, caused by *Trypanosoma cruzi*, remains a significant public health challenge, particularly in Latin America, where it is one of the most neglected diseases and is primarily transmitted by triatomine insects. The disease exhibits complexity due to its diverse transmission routes, including vectorial and non-vectorial mechanisms such as blood transfusions and congenital transmission. Effective monitoring and control strategies are critical to mitigating its impact. This review focuses on current monitoring and control efforts, emphasizing the importance of enhanced surveillance systems, improved risk assessments, and integrated vector control programs. Surveillance plays a pivotal role in early detection and timely intervention, particularly in endemic regions, while vector control remains central to reducing transmission. Moreover, the development of novel diagnostic tools, treatments, and vaccines is a crucial step in advancing control efforts. This review also highlights the involvement of local governments, international organizations, and civil society in executing these strategies, stressing the need for sustained political commitment to ensure the success of public health programs. By addressing key challenges in monitoring, control, and prevention, this review aims to provide insights and recommendations to further global efforts in reducing the burden of Chagas disease.

## 1. Introduction

Chagas disease, also known as “American trypanosomiasis”, is an important zoonotic disease transmitted by vectors to humans, caused by the hemoflagellate protists *Trypanosoma cruzi*. It is part of a group of neglected diseases that primarily affect vulnerable populations and are increasingly expanding their distribution as a consequence of urbanization and migration [1,2]. Its cycle was fully described in 1909 by the Brazilian physician Carlos Ribeiro Justiniano Chagas [3]. Currently, this disease is endemic in 21 countries in Latin America (Argentina, Belize, Bolivia, Brazil, Chile, Colombia, Costa Rica, Ecuador, El Salvador, French Guiana, Guatemala, Guyana, Honduras, Mexico, Nicaragua, Panama, Paraguay, Peru, Suriname, Venezuela and Uruguay), as well as in the United States, particularly in the southern states, where—though rare—estimates suggest it could affect up to 10,000 individuals [4,5,6].

Chagas disease evolves in phases, with the acute phase usually presenting few or no particular symptoms; some patients experience fever, lymphadenopathy, splenomegaly and/or edema, but rarely with severe disease [7]. After the acute phase, individuals infected with *T. cruzi* evolve to a clinically silent phase [7,8]. However, over a period of 10 to 30 years, 20% to 35% of patients develop chronic symptoms of Chagas disease, which is mainly characterized by affecting the cardiovascular, gastrointestinal and/or central nervous systems [8,9]. The remaining infected individuals may persist asymptomatic throughout their lifetime.

According to estimates by the World Health Organization (WHO), which classifies Chagas disease as one of the “neglected tropical diseases”, it is a major public health problem, primarily in Latin America. An estimated 6 to 7 million people could be infected with Chagas disease [5,10], causing 22,000 to 50,000 deaths per year [11,12].

In the last 20 years, many factors have contributed to geographic variation in the burden of Chagas disease [13,14,15,16]. For example, due to increased migration and international travel, Chagas disease is spreading to other geographic areas, mainly in Europe [17], Canada [18,19], New Zealand, and Australia [20]. Estimates suggest that 80,000 to 120,000 immigrants infected with *T. cruzi* live in Europe, and 300,000 live in the United States [21].

Chagas disease is integrated into an eco-epidemiological network, making it one of the most complex human vector-borne diseases [22]. Its causative agent, Trypanosoma cruzi, is a genetically diverse protist capable of infecting a broad spectrum of vertebrates, including humans and more than 100 other mammalian species [23,24,25,26].

This protist is primarily transmitted to its host community through the autoinoculation of contaminated feces from a diverse array of hematophagous insect species within the subfamily Triatominae (Hemiptera: Reduviidae) [27,28]. However, it can even spread by non-vectorial mechanisms, such as blood transfusion [8,29], congenital transmission [8,16], organ/tissue/cell transplantation [8,30], oral transmission because of food contaminated by infected triatomine feces [8,31,32], laboratory accidents [8], vertical or placental transmission, and through the birth canal at birth [33].

To address the complexity of the eco-epidemiology of Chagas disease, it has been essential to implement efficient control strategies that address the factors driving disease risk across spatial and temporal ranges. The establishment of novel control strategies is even more important knowing that there is a growing consensus that the disease is currently a global public health problem and will remain so for the foreseeable future. For example, the development of an effective vaccine or other improved and novel therapies is the next crucial step in the fight against Chagas disease [34,35,36]. It has also been essential to develop sustainable vector control strategies based on improved risk assessment of infection and disease [37,38] or to improve surveillance programs in all endemic regions [14,39]. Finally, another concern of relevance in the effort to combat Chagas disease is to maintain a sufficiently high level of political priority to promote and fund surveillance as well as research to avoid failures in the success of public health programs and a possible evolution of the disease [14,40,41]. The future success of the fight against Chagas disease depends on efficient management of newly emerging infectious foci, maintenance of high levels of public awareness and governmental interest in controlling the disease, and continuous improvements in diagnostic, therapeutic and surveillance tools [42]. The objective of this review is to describe the current status of strategies to control Chagas disease and highlight new research topics to reduce the burden of this public health problem.

Specifically, we address selected aspects that include the biology of *T. cruzi* to better understand the dynamics of its transmission, the identification of methods focused on the prevention and control of the disease developed by local governments, international organizations, public health and civil society with both scientific and traditional or homemade approaches, as well as the recognition of the main social and ecological factors that have historically played a role in favoring or reducing the presence of the disease vector in the Latin American region. Altogether, this will serve as a basis for proposing recommendations directed under an integral vision.

## 2. Review

### 2.1. Trypanosoma cruzi Taxonomy

The taxonomy of *Trypanosoma cruzi* remains a subject of ongoing debate, with increasing genomic and molecular evidence challenging traditional classifications. Currently, *T. cruzi* is divided into six discrete typing units (DTUs), TcI to TcVI, based on multilocus sequence typing and phylogenetic analyses. However, recent studies suggest a more complex population structure with high genetic diversity and potential hybridization events [43]. In this context, the development of a comprehensive phylogenetic framework for the Trypanosomatidae family, as proposed by Kostygov et al. [44], is crucial for exploring trait evolution and refining taxonomic classifications. Advances in whole-genome sequencing have significantly expanded our understanding of *T. cruzi’s* genetic variability, particularly through large-scale genomic projects that aim to elucidate the mechanisms underlying its high genetic plasticity and host adaptation [45]. Comparative genomics studies have revealed extensive gene copy number variations, repetitive sequences, and large segmental duplications, which contribute to the parasite’s adaptability and differential virulence among DTUs [46]. Moreover, transcriptomic and epigenomic approaches have provided insights into gene regulation and host–parasite interactions, highlighting the role of gene expression variability in pathogenesis and transmission cycles [47]. With recent technological advances, including long-read sequencing and single-cell genomics, ongoing projects continue to refine our understanding of *T. cruzi* diversity and its epidemiological implications [48].

A clearer understanding of *T. cruzi* taxonomy has direct implications for parasite transmission dynamics, diagnostics, and treatment strategies. The DTUs differ in their geographic distribution, host range, and clinical outcomes. For example, TcI is the most widely distributed DTU in the Americas and is associated primarily with synanthropic transmission cycles and chronic cardiomyopathy in humans [49]. In contrast, TcII and TcV are predominant in the Southern Cone and are linked to severe cardiac manifestations of Chagas disease [50]. Moreover, TcIV, which circulates in both North and South America, has been implicated in atypical Chagas disease cases, including oral transmission outbreaks in the Amazon region [51]. DTU distribution is also closely linked to ecological differences in host use and habitat preferences; for instance, TcI is frequently found in marsupials and armadillos in sylvatic cycles, whereas TcII, TcV, and TcVI are more commonly associated with a great variety of terrestrial mammals, including synanthropic rodents and domestic animals, facilitating their transmission in human environments [23]. These ecological associations not only shape the parasite’s geographical distribution, but also influence the epidemiological risk for humans by determining the likelihood of spillover from wildlife to domestic transmission cycles. Understanding these host–parasite relationships is critical for designing effective control strategies as reservoir host diversity directly affects transmission dynamics and human infection risk.

DTU-specific differences also impact diagnosis and treatment. Variability in antigenic profiles between DTUs affects the sensitivity of serological tests, sometimes leading to false-negative results, particularly in cases involving TcI or hybrid DTUs such as TcV and TcVI [52]. From a therapeutic perspective, susceptibility to benznidazole and nifurtimox is known to vary among DTUs, with TcI strains often exhibiting higher resistance compared to TcII and TcVI [53]. Such variations highlight the need for DTU-targeted treatment approaches and the development of new chemotherapeutic options. Additionally, the application of omics tools, including cytogenomics and genomics, to the study of Chagas disease vectors has enhanced our understanding of vector biology and taxonomy, which is essential for developing effective control measures [54]. These findings emphasize the necessity for a more dynamic and integrative classification framework that accounts for the parasite’s genetic complexity and potential cryptic diversity.

### 2.2. Vector Transmission

The transmission of *T. cruzi* in animals and humans occurs through hematophagous insect vectors whose contaminated feces come into contact with bite sites or mucous membranes. Currently, 144 species of triatomines have been described, with firm evidence that more than a half of them can carry *T. cruzi* [11]. Triatomines belong to the Reduviidae family with the most epidemiologically important genera being *Rhodnius*, *Triatoma* and *Panstrongylus*, also known as “kissing bugs” [32,55]. Although the transmission of the disease is mostly associated with triatomines in domestic habitats, some wild triatomine species have a relevant role in the transmission of *T. cruzi* to humans, especially in rural and peri-urban areas [41]. However, only a limited number of species are associated with environments close to humans and are considered of medical importance [56].

*Triatoma infestans* and *Rhodnius prolixus* are the most efficient and relevant vectors in the transmission of *T. cruzi* due to their tolerance to humans and their high adaptability to households. These two species vary in their habitats, between sylvatic, peri-urban and domestic environments. *T. infestans* and *R. prolixus* are predominantly domestic, although both species maintain sylvatic populations. In contrast, other species, such as *T. dimidiata* and *P. megistus*, are primarily sylvatic, with some populations exhibiting human tolerance or a high degree of domestication [14]. Triatomines feed mainly on the blood of humans, birds or mammals, although there are some reports that mention that some species can even feed on the blood of reptiles or amphibians. They have an incomplete metamorphosis since development includes the egg stage, five nymphal stages and adults, thus being considered hemimetabolous insects [57]. The nymphal stages include five developmental phases before reaching adulthood, characterized by a progressive increase in size and blood-feeding capacity. Nymphs emerge from the egg during the first stage, being small and vulnerable, feeding only once before molting. In subsequent stages, they grow larger and more mobile (second and third stages), acquiring a greater ability to feed on blood. During the fourth stage, nymphs reach a size close to that of adults, but lack sexual maturity. Finally, in the fifth stage, known as the pre-adult stage, they prepare for the final molt, during which they develop functional wings and fully formed sexual organs. These stages have variable durations depending on the species, temperature, and food availability [58,59].

Triatomines require at least one blood meal during the five nymphal stages in order to continue their development. The female may deposit hundreds of eggs during her lifetime. These eggs hatch approximately 10 to 30 days later, depending on the temperature and humidity of the environment [14]. Generally, triatomines feed during the night, although, under conditions of food stress, they may come out during the day in search of food. In laboratory-reared colonies, triatomines feed equally during the day and at night [55].

After feeding, they defecate near the wound, and if the host scratches or presents an open lesion, the parasite present in the insect’s feces or urine can enter the body through the skin or mucous membranes, causing infection [60]. The main Latin American vectors, such as *T. infestans*, *R. prolixus*, *T. dimidiata*, *P. megistus*, *T. brasiliensis* and *T. sordida* and *R. pallescens*, defecate during or immediately after ingesting blood. It has been reported that most sylvatic triatomine species can colonize the nests of their food sources, establishing close associations with rodent and marsupial species, which are recognized as potential wild hosts. These hosts frequently move between human dwellings and forested areas, acting as reservoirs that facilitate the movement of *T. cruzi* between the sylvatic and domestic environments [61].

Population growth, migration and the high concentration of humans have seriously affected the natural mechanisms for vector control, related to environmental transformations, including factors such as climate change, land use change, uncontrolled urbanization and deforestation [62]. These have favored the redistribution and adaptation of Chagas vectors to new areas and environments and consequently led to an increase in the risk of parasite transmission to humans [63]. Therefore, vector control has become a challenge because its presence depends on social, biological and environmental interactions [60,62,64]. Both environmental impact and socioeconomic inequity drive parasite infection rates, and may determine its distribution and the mortality it causes [61].

In line with the above, the presence of the principal vectors of Chagas disease pathogens in people has historically been linked to dwellings established in rural areas. This type of domicile has been the main target for the detection of vector infestation processes. However, based on the multiple impacts caused at the ecosystemic level, which have consequently affected the natural dynamics of vectors, more and more urban and peri-urban infestations are being reported [65].

Therefore, traditional vector control and prevention programs have focused on the eradication of these organisms in rural dwellings due to their physical vulnerability. However, vector control programs now also exist at the urban level. In both rural and urban areas, home improvements at the infrastructure level are promoted, as well as health education, manual collections and mainly the use of chemical insecticides [66].

Poverty and infrastructure conditions promote the permanence of the vector because the homes, generally infested, are usually built with precarious materials (adobe, wood, thatched roofs) that have cracks or holes in which triatomines can hide and reproduce, as well as dirt floors [67]. These conditions provide them with viable shelters for nesting, which is why control and prevention methods suggest focusing on improving housing conditions, which can be classified mainly into physical and chemical methods, without leaving aside other methods such as biological control and genetic control [66].

#### 2.2.1. Traditional or Home-Based Vector Control and Prevention Methods

Traditional or home-based methods have long been used by society, particularly in rural and indigenous communities, to prevent infestations of disease vectors such as insects and small mammals. These practices, rooted in local knowledge, include both physical and chemical approaches applied inside or around dwellings based on household needs. While their effectiveness may vary and often lacks comprehensive scientific validation, they remain valuable preventive measures, especially in areas with limited access to formal control programs [68,69,70,71].

One widely used approach in rural localities involves the application of insecticide sprays, which serve as a practical and accessible solution. Additionally, preventive measures emphasize household cleanliness, such as plastering floors, removing accumulated materials outside the house, and maintaining clean yards and corrals to minimize breeding sites for insects. These measures reflect a cultural association between vector presence and household hygiene [68,70].

Likewise, in Latin American rural communities, the role of women in vector control and insect prevention in the home is highlighted as they are responsible for carrying out these measures. For example, in the Guaraní indigenous communities of Gutiérrez, Bolivia, women play an active role in the prevention and control of the disease, highlighting that they are primarily responsible for carrying out the cleaning of homes, mainly the yard and corrals, in places identified as appropriate for vectors to inhabit [66]. In turn, women play a central role in household insect control measures in the villages of Bokoba, Teya, and Sudzal, located in the central part of Yucatan, Mexico, driven primarily by their motivation to protect their children. Their proactive efforts, including spraying insecticides, sealing windows and doors, and employing repellents, reflect their pivotal contributions to family health and well-being [70]. These traditional methods, while sometimes viewed as supplementary to formal control strategies, highlight the ingenuity and resourcefulness of communities in safeguarding their health and environments.

#### 2.2.2. Physical Methods

Home improvement is one of the most effective ways to reduce the vector population, especially in rural areas. Examples include plastering walls and sealing crowded spaces as the vector hides in cracks and gaps in walls and ceilings; so, sealing walls and ceilings helps reduce its habitat. The type of roof and wall material is important, not because of the material itself, but because of the way it is used; adobe, cane and mud or stick and pique can enable the appearance of cracks that serve as protection for the vector. The same occurs with thatched or reed roofs [72].

Kissing bugs, due to their naturally nocturnal habits, tend to prefer dark environments, and thus lighting in homes with warm or blue lights, by keeping some lights on outdoors at night, while not eliminating the problem, may help deter these [73]. In contrast, another physical method of control is homemade UV or CO_2_-emitting light traps. These are traps that are made from simple materials (such as plastic bottles) and emit CO_2_ by mixing yeast and sugar, which attract bed bugs, as does UV light. Triatomines are attracted to certain wavelengths of light, especially in the ultraviolet (UV) spectrum. Light traps emit light in this range, which causes insects to approach and become trapped. These traps are especially useful in dark areas or at night, when insect activity is highest. Light traps are popular in endemic areas because they allow vectors to be captured economically and without pesticides [73]. Although these traps do not eradicate the infestation, they can help monitor the presence of these insects and reduce their proliferation.

Thermal control is also an important element in vector control since the triatomine bug is sensitive to extreme temperatures, and thus applying intense heat to places where they live can eliminate larvae and adults. *T. infestans* is able to tolerate a minimum temperature close to 0 °C, while *R. prolixus* does not tolerate temperatures below 5 °C. However, the difference is smaller at the maximum temperature, as both tolerate up to approximately 50 °C [73,74]. However, this is less common in mass control campaigns due to the difficulty of applying it effectively [73].

#### 2.2.3. Chemical Methods

Although household insecticides (such as common insect sprays) are not as effective as residual insecticides, they appear to help if applied regularly to specific locations where triatomine bugs may hide [66,75]. There are sources of plant origin that seem to be useful for the control of kissing bugs, such as essential oils with repellent function, and there is knowledge of the effective toxicity of eucalyptus and neem (*Azadirachta indica*) oils on triatomine bugs [66]. Another example is cinnamon oil, which inhibits molting, and citronella oil, which causes insects not to feed, leading to their premature death. This is because plants produce secondary metabolites, many of which have an insecticidal or repellent effect. For example, the monoterpenes eucalyptol and geraniol have lethal effects on some species of kissing bugs. Other compounds, such as carvacrol, carveol, citronellol, eugenol, linalool, menthol, a-terpineol, thymol and verbenol, have repellent effects against triatomine bug nymphs [66,76].

Due to the volatile property of essential oils, they may have poor environmental stability, leading to a short period of effective control and subsequently increasing the cost of triatomine control [76]. Cleaning with vinegar and water solutions appears to be another effective home control method as vinegar acts as a repellent due to its odor and acidity. Acidic components, such as acetic acid, generate a strong odor that is unpleasant to many insects, disrupting their ability to detect olfactory cues, such as carbon dioxide or sweat, which often attract them to humans or animals [77].

The use of insecticides that have a prolonged effect, applied on walls, ceilings and cracks in and around dwellings, is common. Residual-action insecticides act for weeks or even months after application, eliminating bedbugs that come into contact with treated surfaces [78]. Some of the most common compounds are pyrethroids, such as deltamethrin and permethrin. They are effective and have low toxicity to humans and domestic animals, being the most widely used insecticides in vector control programs [75]. Organophosphates such as malathion, although their use has decreased due to their higher toxicity and lower residual effectiveness compared to pyrethroids [75], have historically also been used. Complete fumigation of dwellings and adjacent structures is especially useful for treating large areas in a short time but requires that people and domestic animals be temporarily absent from their homes to avoid exposure [78].

Although the use of baits is the least common method in eradication campaigns, some bedbug traps use insecticides in the baits. When the vectors are attracted and come in contact with the baits, they die from poisoning. This method is more common in research or as a control measure in small areas [79]. Outdoor fogging is recommended only in situations of high risk for disease transmission, where the intensity of the infestation is very high or uncontrollable by traditional methods and the vulnerability of the community justifies the use of such an intensive technique [70].

In recent years, there has been increasing research on the use of entomopathogenic fungi, nematodes, viruses, endosymbionts, and parasitoids as biological control of vectors [75,80]. Biological control of a vector refers to the use of living organisms, such as predators, parasites, pathogens, or competitors, to reduce the population or limit the ability of a vector to transmit disease [41]. The use of two fungal agents that have exhibited mortality in the nymphal stages of the triatomine bug has been proposed. Metarhizium anisopliae and *Ixora javanica* have been widely used as effective biological control agents with chinch bugs in agricultural environments [80]. Both entomopathogenic fungi are an effective alternative to control adults and immature stages of one of the main vectors of Chagas disease [80].

Research on the effects of entomopathogenic nematodes against triatomines is limited. Entomopathogenic nematodes have an almost unique combination of desirable attributes that make them successful bioregulators [81]. Two nematode genera, *Heterorhabditis* and *Steinernema*, in a nematode–bacterium complex (*Photorhabdus luminescens*), have demonstrated the ability to kill *T. infestans* and *R. prolixus* under laboratory conditions [75,81]. Heterorhabditis indica could be a viable biological control for adults of *T. dimidiata*, especially in areas with resistance to chemical insecticides. However, further studies are needed to evaluate its effectiveness and specificity under field conditions and its impact on non-target insects [82]. *Triatoma* virus (TrV) is a non-enveloped virus of the family Dicistroviridae [83,84]. Although there is no evidence to suggest that TrV suppresses *T. cruzi* in triatomines, it can induce high mortality in *T. infestans* and subsequently reduce populations, which is promising for use as a biological control for triatomines and Chagas disease [75].

Endosymbionts as genetic control is another way to achieve vector population control or make populations refractory to the transmission of disease-causing agents [75]. Some entomopathogenic endosymbionts have shown potential, mainly nematode-associated bacteria, such as *Photorhabdus luminescens* and *Xenorhabdus* spp., which live in symbiosis with nematodes of the families Heterorhabditidae and Steinernematidae. These endosymbionts release toxins into the vector’s digestive system, as in the case of *T. infestans*, producing high mortality [85]. The field release of genetically modified bacteria is risky. An additional barrier to the implementation of this intervention is the possibility of undesirable environmental impacts. Paratransgenesis can threaten the diversity of the microbiome and influence horizontal gene transfer [75]. Parasitoids are organisms, which spend a significant part of their life cycle in or on the body of a host, eventually causing its death, and unlike parasites, do not cause the death of the host [73,86]. Multiple parasitoid species of triatomines have been reported, such as *Telenomus fariai Costa Lima* (Hymenoptera: Scelionidae), *T. costalimai*, *Megaselia scalaris* and *Aprostocetus asthenogmus* [75].

In this context, the fight against the vector of Chagas disease, such as *T. infestans* and other triatomine species, is generally led by international agencies such as the World Health Organization (WHO), Pan American Health Organization (PAHO), Drugs for Neglected Diseases Initiative (DNDi), national governments, public health organizations and community initiatives that conduct specific programs for the control and surveillance of Chagas disease [87,88,89,90,91,92,93]. An example of this is the formalization of epidemiological and entomological surveillance activities for Chagas disease at the national level through a program governed by the Ministry of Public Health in Ecuador [15]. These programs are crucial for the development of surveillance activities and provide insight into the current situation of countries in the fight against neglected diseases such as Chagas disease.

However, vector-borne transmission of Chagas disease has not been effectively controlled in many regions of Latin America, and the application of strategies to suppress vector populations has not been fully effective. A case study developed in the Gran Chaco ecoregion, Argentina, through community spraying and intensive monitoring for about 3 years shows that selective insecticide spraying, previously recognized as an effective method of control, did not perform efficiently in the detected vector foci. Therefore, the authors highlight the need to improve not only management and surveillance actions at the household level, but also to reduce the operational, economic, and political constraints that contribute to increase habitat suitability for the vector [94].

Current control programs in Latin American countries have focused on this method together with health education for the interruption of domestic and peridomestic cycles of transmission involving vectors [95]. A success story is the control program implemented in Brazil since 1975, which has led to a significant reduction in the *T. infestans* vector, with only 485 insects nationwide in 1998 [95]. However, although much of the Southern Cone countries have focused their efforts on the surveillance phase and focal spraying, when necessary, several differential factors converge to maintain the infestation of dwellings and consequently the transmission of the disease [93,95], such as re-infestation of dwellings by triatomines [91], resistance to pyrethroid insecticides in *T. infestans* [93,96], and non-vectorial transmission [8,16,29,31].

### 2.3. Non-Vector Transmission

Chagas disease can also spread by non-vectorial mechanisms, such as blood transfusion [8,28], congenital transmission [8,16], organ/tissue/cell transplantation [8,30], food contaminated by infected triatomine feces (oral transmission) [8,31], laboratory accidents [8], vertical or placental transmission, and through the birth canal at birth [33]. Blood transfusion and the transplacental or breast milk route are the main transmission mechanisms in non-endemic countries [57]. The risk of blood transfusion has decreased due to screening in all blood banks in Latin American countries [97].

Maternal–fetal transmission of *T. cruzi*, also known as vertical transmission, occurs when live parasites persist and multiply in the fetus or after birth, leading to congenital infection, and can take place prenatally (in utero), perinatally (during delivery), or postnatally (after birth) [98]. A possible mode of maternal–fetal transmission could be through parasites released in the amniotic fluid. The latter could contaminate fetuses via oral or pulmonary routes, or eventually by cutaneous penetration (fetuses bathe in amniotic fluid and continuously absorb it) [98]. Transmission through contact with equipment or handling of materials infected with viable parasites in research or clinical laboratories may represent a non-vectorial source of risk for human infection with *T. cruzi*. Because they can occur in many circumstances and can often go undetected or undiagnosed, laboratory-acquired cases of *T. cruzi* infection are likely to be underreported [98].

Finally, oral transmission of *Trypanosoma cruzi* has emerged as a significant route of Chagas disease transmission, particularly in regions where vector control measures have reduced traditional vector-borne transmission. The primary causes of oral transmission include the ingestion of contaminated food or beverages containing *T. cruzi*-infected triatomine feces, crushed triatomine insects, or contaminated secretions from infected mammalian reservoirs [31,99]. This mode of transmission is most frequently reported in the Amazon and other forested areas of South America, where the parasite is maintained in complex sylvatic cycles involving a diverse range of wildlife reservoirs and vector species [100]. Freshly prepared fruit juices, particularly açaí pulp, sugarcane juice, and other unprocessed food items, have been implicated in multiple outbreaks, highlighting the role of food hygiene and processing in disease prevention [101,102].

Poor sanitary conditions, inadequate food handling practices, and limited awareness contribute to the risk of contamination, particularly in endemic rural and peri-urban areas [103]. Additionally, oral transmission can occur through the consumption of raw or undercooked meat from infected wild mammals, including marsupials and rodents, which serve as natural reservoirs for *T. cruzi* [104].

A comprehensive review identified 32 outbreaks of orally transmitted Chagas disease between 1965 and 2010, with a notable increase in cases reported between 2006 and 2010 [105]. These outbreaks, primarily occurring in Brazil, Colombia, and Venezuela, underscore the growing importance of oral transmission in the epidemiology of Chagas disease. The rising frequency of oral transmission cases emphasizes the need for enhanced surveillance, food safety regulations, and public health campaigns to mitigate this overlooked yet growing route of Chagas disease transmission.

#### Methods of Control and Prevention of Non-Vector Transmission

The prevention of non-vectorial transmission of Chagas disease requires strategies to minimize the risk of infection through alternative routes, such as blood transfusions and organ transplants. Mandatory screening of blood and organ donations using specific tests to detect *T. cruzi* is essential in endemic regions and countries receiving migrants from affected areas, as it ensures the exclusion of positive donors, significantly reduces the risk of transmission, and requires careful monitoring of both donors and recipients in organ transplants [106]. Recent studies indicate that the implementation of comprehensive screening programs in healthcare systems has nearly eliminated the transmission of *T. cruzi* via transplantation in structured settings [106,107]. These preventive measures, combined with community education and awareness, play a vital role in controlling the spread of Chagas disease [5,98].

In the case of congenital transmission, early diagnosis in pregnant women, especially in high-prevalence areas, is essential. Timely treatment of infected neonates is highly effective and prevents long-term complications [98]. Women of childbearing age from endemic regions should undergo screening, and antiparasitic treatment should ideally be completed before pregnancy to reduce the risk of fetal infection [5]. Neonatal screening is equally important for early detection and treatment in infants born to infected mothers. Examining neonates from infected mothers using PCR or microhematocrit tests ensures the early identification and treatment of infections, which is crucial for preventing disease progression [107,108]. Moreover, follow-up care for children of HIV-positive mothers ensures the identification and management of vertically acquired infections [96]. Both benznidazole and nifurtimox are highly effective when administered during the first year of life for treating congenital Chagas disease [32]. The effectiveness of these interventions is supported by evidence indicating that treatment prior to pregnancy reduces congenital transmission nearly to 100%, while neonatal screening and prompt treatment in infected infants achieve similar rates of cure [5,106].

Consumption of food or beverages contaminated with infected material, although usually less frequent, represents another important avenue of prevention. Preventive measures include ensuring proper food hygiene, the use of safe drinking water, along with vector control in food production areas, and the safe processing of products such as cane or fruit juices, particularly in rural areas of Latin America. Maintaining strict food and beverage hygiene, coupled with public awareness campaigns, has proven effective in reducing cases. [109].

Finally, in laboratory settings, the implementation of strict biosafety standards and specialized training for handling potentially infectious samples significantly reduces the risk of accidents that can result in occupational transmission. These measures include the use of appropriate personal protective equipment, standardized procedures for handling and discarding contaminated material, and ongoing training of personnel in safe practices [110]. In addition, strengthening health systems through community education and access to modern diagnostic resources is essential to address non-vectorial transmission comprehensively and effectively [109]. These strategies not only decrease the incidence of new infections, but also contribute to a broader control of Chagas disease in diverse epidemiological contexts.

### 2.4. Host

As part of the cycle, in addition to the vector, the host plays a fundamental role in the maintenance of the disease. The relationship between *T. cruzi*, the causative agent of the disease, and its hosts is fundamental to understanding the epidemiology and transmission of the disease.

The hosts of Chagas disease are diverse and include both wild and domestic animals, as well as humans [3]. In the domestic cycle of the disease, the main hosts include dogs, cats, farm animals (cattle, sheep, goats, pigs) and poultry [67]. Dogs and cats are easily infected and can harbor the parasite in the bloodstream, allowing triatomines to become infected by feeding on them [110]. Dogs are considered the most important peridomestic reservoirs as they develop symptoms similar to humans and can increase the risk of transmission if they are in close contact with bed bugs inhabiting dwellings or nearby areas.

Infection in cats has also been identified, although it is less studied [111]. Farm animals can be infected by *T. cruzi*, especially in rural areas where these animals coexist with triatomines. While their role in direct transmission to humans is minor, they contribute to the transmission cycle by enabling the feeding of infected and uninfected triatomines [41]. Although birds are not direct hosts of *T. cruzi*, because the protists cannot survive in them, they may play an indirect role. Triatomines often feed on birds, which allows them to survive and thrive in rural and urban areas. In this way, birds can help maintain triatomine populations near homes and in contact with other animals that can be infected [41].

To date, 37 species of wild mammals belonging to the orders Marsupiales, Cingulata, Edentata, Chiroptera, Carnivora, Arthiodactyla, Rodentia and Primates have been confirmed positive for the disease. These include animals inhabiting sylvatic or peridomestic environments, including rodents, raccoons (*Procyon lotor*), and opossums (*Didelphis virginiana*), as well as Ocelots (*Leopardus pardalis*), coatis (*Nasua narica*) [4], and bats [112,113]. These animals act as key reservoirs, as the parasite can persist in their organisms without causing them great effects, thus facilitating transmission through hematophagous vectors that infect them and then transmit the parasite to other hosts, including humans [41].

Since rodents are at the base of many food chains and inhabit a wide range of environments, they are critical links between the sylvatic and domestic transmission cycle of *T. cruzi*. Their importance is due to biological, ecological and behavioral factors, which make them efficient reservoirs in different environments, making them relevant species for understanding the transmission dynamics of the disease and foci of attention in control and surveillance programs [114]. Due to the limited availability of studies on infection in wildlife, the role of *T. cruzi* may be underestimated. Although wildlife species play important roles as sylvatic reservoirs, investigations of *T. cruzi* pathology in wildlife are limited to a few studies documenting histological lesions in opossums and raccoons [115]. It has been found that infected animals such as coyotes (*Canis latrans*) can show cardiac inflammation; however, it has not been attributable to Chagas disease, which may lead to severe underestimation due to the existence of unknown mammalian hosts [94].

### 2.5. Experimental Drugs and Plant-Based Drugs for Treatment and Prevention

Experimental drugs are pharmaceutical compounds that have been authorized by the United States Food and Drug Administration (FDA) for human testing in specific conditions but have not yet received approval for commercial distribution or widespread use. These drugs undergo rigorous evaluation through clinical trials, which are structured research studies involving human participants to assess their safety, efficacy, and potential therapeutic benefits. If the outcomes of these trials confirm their safety and effectiveness, regulatory agencies may approve them for market release [116,117]. Similarly, experimental vaccines are immunological preparations that are still under investigation to determine their capacity to induce protective immunity against specific pathogens. These vaccines progress through preclinical and clinical phases before they can be approved for general use. On the other hand, plant-based drugs are therapeutic agents derived from natural plant sources, utilizing bioactive compounds identified through traditional medicine and scientific research. These compounds have demonstrated potential for treating various diseases, including neglected tropical diseases such as Chagas disease, by targeting key biological pathways involved in pathogen survival and host–pathogen interactions [118].

#### 2.5.1. Dewormers (Human and Animal)

In some regions, domestic animals, such as dogs, have been found to play an important role as a reservoir of the parasite and its transmission to humans [36,119]. Therefore, infection control in these animals has been proposed as an important complementary strategy to vector control, for example, by using collars impregnated with insecticides such as deltamethrin (DTDC) [120], and topical application of the combination of fipronil and permethrin to vector-exposed dogs [121]. The topical formulation of imidacloprid was effective against pyrethroid-resistant *T. infestans* populations when applied to pigeons [122] at the laboratory level. Likewise, xenointoxication has been suggested for triatomine control [123], with products such as ivermectin applied to dogs [75,124], cypermethrin in poultry chickens [125,126] and the use of fluralaner for vector control in both chickens and dogs [126,127,128,129].

In the treatment of Chagas disease at the human level, two drugs are currently used: Nifurtimox and benznidazole, effective for the acute phase [130] and congenital cases [131]. Benznidazole (which acts by damaging the parasite’s mitochondrial DNA) and Nifurtimox (which induces oxidative stress, inhibiting parasite growth) are the only drugs with proven efficacy [58]. However, in recent years, it has been demonstrated that benznidazole, in the treatment of patients with chagasic cardiomyopathy, can work very well in curing patients during the early stages of the disease, especially newly infected children and young adults [132].

#### 2.5.2. Vaccination Projects

The first attempts to develop a Chagas disease vaccine started by inoculating mice with strains of *T. cruzi* in 1913, just four years after the discovery and report of the parasite causing the disease [133]. In these assays, the mice received only partial immunity, with a reduction in mortality rate but persistent parasitemia. Since then, the development of a vaccine for Chagas disease has faced several challenges, and some of the main issues to be addressed (but not limited to) include the complex parasite life cycle (different stages and multiple forms of the parasite), a limited understanding of the protective immunity mechanisms (precise immune response required to protect against the disease is not fully understood), a lack of predictive animal models (mice do not reflect the human disease, dogs are better models, but may not fully recapitulate the dynamics of the disease in humans) and limited commercial interest (primarily affects marginalized and economically disadvantaged populations in Latin America) [36,134,135,136].

Vaccines have been developed to date, including trials of attenuated vaccines, DNA vaccines, viral vectors, recombinant protein or peptide vaccines, glycoconjugates, multivalent vaccines, heterologous vaccines and mRNA vaccines [137,138,139,140]. An extensive and very comprehensive comparison amongst the known vaccine trials, phase of the disease analyzed, time of vaccine initiation in the case of therapy, the animals used, the strain of *T. cruzi* and doses used, and the level of significance observed for each outcome was reviewed first by Quijano Hernández and Dumonteil [36], and updated by Farany and collaborators [135].

Given the need to improve the treatment of the disease and provide timely care of the infection, the search for new drugs and, more recently, the development of vaccines that are effective for its control at all levels of infection caused by *T. cruzi* has been crucial. It has been estimated that the advantages of having a vaccine that prevents Chagas disease are significant not only in terms of public health, but also in terms of economic and social development [98]. In this sense, the development of proposals for the creation of vaccines has been a significant advance but so far limited to a few emerging studies. Nevertheless, these findings have allowed for the recognition of certain characteristics and chemical compounds of importance, which provide a positive response for the prophylactic treatment of Chagas disease.

Among these advances, the development of vaccines could help control the infection and pathology of the disease [141]. For example, it has been recognized that anti-*T.cruzi* vaccines should have certain characteristics at the molecular level that make them suitable candidates for incorporation. These characteristics include being expressed in all stages of *T. cruzi*, being conserved and expressed in its different strains, and being crucial for the development and survival of the pathogen [139,142]. In this scenario, the cruzipain enzyme has been considered a target for developing prophylactic treatment because it is found within the three main developmental stages of *T. cruzi* in all strains that have been tested, thus being crucial to achieve internalization into host cells [143]. Likewise, the development of an effective vaccine against *T. cruzi* must consider the high levels of genetic variability in these protists due to the restrictions in protective efficacy reported in some strains [144,145] that may disrupt vaccine efficacy.

Studies at the molecular level have led to an important basis in the recognition of other key molecules such as the Tc52 molecule, a glutathione S-transferase for the development of prophylactic vaccines, since this molecule is vital for the survival of *T. cruzi* as it is expressed during all phases of development, although mainly in the replicative stages: epimastigote and amastigote. Immunization based on this molecule enabled the production of anti-Tc52 antibodies, thus showing defense against infection [139]. In addition, it has been evaluated that the enzyme prolyl oligopeptidase Tc80 could be considered another candidate to be used as a prophylactic since it is expressed in the blood and amastigote trypomastigote, allowing for invasion into host cells as it participates in the degradation of the extracellular matrix, collagen and fibronectin [137]. The use of this molecule elicited a strong immune response both humoral and cellular since anti-Tc80 antibodies caused enzymatic inhibition and lysis of trypomastigotes by activating the complement system [132].

##### Who Is the Vaccination Aimed at? Examples of Animal Experiments

In line with the above, the development of preventive and therapeutic vaccines against *T. cruzi* evaluated in mice has led to the recognition and testing of various formulations, mainly based on the application of whole organisms (live attenuated *T. cruzi* and live *T. rangeli*), targeting reservoir species as a means to alleviate the burden of disease [146,147], recombinant proteins that induce immunity [143,148,149,150,151,152,153,154], viral vectors [155,156,157], heterologous primary drivers [158,159], and DNA [148,160,161,162,163]. These findings have shown diverse results in the immune response and parasitemia of the disease; in most cases, survival was increased and, in a few cases, it was variable according to the challenge route [36].

Certain therapeutic vaccines have also been evaluated very recently in rhesus macaques [161] and historically in mice [164]. Particularly, disease development in dogs presents a much more similar evolution to that in humans [36,134,165]. Therefore, dogs have been considered candidates to evaluate these vaccines [36]. The main formulations evaluated include recombinant proteins and DNA [162,166,167,168]. However, a single DNA-based formulation with the TSA-1 antigen, Tc24 and Tc52, has resulted in a decrease in parasitemia and parasite load in the heart, with an increase in survival [162].

The immunization of mice with heterologous Tc24 mRNA protein is a new approach based on a new generation of RNA-based vaccines that have demonstrated the ability to induce protective immunity, inducing strong antigen-specific CD8+ T cell responses and effective CD4+ T cell responses [146]. In this study, it was possible to verify that heterologous mRNA protein vaccination with Tc24 mRNA to prime and Tc24-C4 protein (a genetically modified cysteine-free polypeptide construct) to stimulate promotes a cellular immune response against *T. cruzi*, characterized mainly by an increase in the level of polyfunctional CD8+ T cells [169].

Another prevention strategy is the development of a canine vaccine to control the transmission and incidence of Chagas disease in endemic countries [170,171,172,173,174,175,176]. Given that dogs have been identified as a key host for the parasite and peridomestic reservoir of Chagas disease, they play an important role in pathogen transmission to humans in several countries and epidemiological settings [110,169,177,178,179].

In Argentina, the application of a vaccine with attenuated parasites to domestic dogs and two boosters every two and fourteen months, demonstrated, by parasitological evaluation by xenodiagnoses, that vaccination reduced natural infection by *T. cruzi* from 26.7% to 12.3% after one year [170]. Also, in a trial with DNA vaccines, the authors found that the prophylactic effect of DNA vaccines induced moderate protection in immunized dogs by preventing cardiac chamber enlargement, poor contractility and heart failure [180]. Despite attempts in the development of a vaccine against Chagas disease, the results, which mainly focus on the immune response and cardiac damage, have been varied; however, none have prevented infection and the degree of protection has been mild to moderate [168,172,174,181].

##### Vaccines Linked to Chemotherapy

The current elective treatment of Chagas disease is oral benznidazole (BNZ), which is most effective in the acute phase of disease. However, BZN has dermatologic and neurologic side effects, and BZN can also produce liver toxicity due to the formation of reactive metabolites, which can damage liver cells, leading to liver injury [135]. A new approach to treat Chagas disease is the use of vaccine-linked chemotherapy, combining the antiparasitic drug with the vaccine antigen. This enables a reduction in the parasite load and an increase in the immune responses in the patient [182,183]. The studies focused on vaccine-linked chemotherapy evaluated the recombinant protein vaccine Tc24-C4 + TLR4 adjuvant in mice and showed a reduction in parasite burden and reduction in cardiac inflammation and fibrosis [182,183,184]. This strategy has been tested on acute and chronic infection models using the H1 strain of *T. cruzi.* This strategy allowed for a reduction in the dose of BZN, which will benefit the patients by minimizing the side effects of the treatment. The test of this regimen in a chronic model of infection showed improved cardiac structure and function and cardiac metabolism [153]. Similarly, the recombinant protein vaccine antigen TS + ISPA was evaluated in conjunction with the curative dose of BZN and showed a reduction in parasite burdens and reduced cardiac arrhythmias. The studies showed that combining vaccine-linked chemotherapy enhanced efficacy in BNZ treatment and offered a dose-sparing approach that may improve overall cardiac, liver health and clinical outcomes.

### 2.6. Environmental Factors Associated with Human Transmission

Chagas disease is endemic in vectors and wild reservoirs throughout the Americas, having spread from the southern half of the United States to Argentina. To date, three main types of transmission cycles for *T. cruzi* have been described, including the sylvatic, domestic, and peridomestic cycle. The domestic cycle is characterized as being maintained or established between interactions by humans, domestic animals such as rats, dogs, and their vectors, mainly triatomine bugs that have adapted to human dwellings [185]. This cycle is considered the most important for maintaining the circulation of the pathogen in human populations because it has concentrated the disease burden in human communities [186].

#### 2.6.1. Sylvatic vs. Domestic Cycle and Its Relationship with the Environment

The sylvatic cycle involves wild mammals such as rodents and marsupials that are infected by individuals from wild triatomine populations [187]. This sylvatic transmission cycle includes raccoons, opossums, skunks, and wild and captive neotropical nonhuman primates from the New and Old World. Considering that this cycle has not been evaluated as important in the generation of human infections, current environmental changes (deforestation, agricultural and livestock expansion) are altering the dynamics of *T. cruzi* transmissions.

There is a type of cycle that has been considered a bridge between the other two cycles of sylvatic and domestic transmission. This cycle arises as a result of the origin of sylvatic transmission of the infection and is maintained by domestic animals in areas close to human dwellings through the action of peridomestic triatomines (for example, dogs and cats that hunt wild animals or wild animals that enter areas close to human dwellings) [188].

Recent studies have shown that there is a direct relationship between the impact of deforestation on ecosystems and the incidence of the disease [1], the favoring of wild vector populations [189], disease prevalence, and its association with vector abundance [190,191]. On the other hand, the impact on natural ecosystems generated by changes in land use has also led to effects on host infection rates, mainly rodents [192]. In particular, a predictive study developed in Mexico on the potential changes in the range of disease transmission cycles as a function of changes in land use, land cover and climate change shows a significant expansion of the range of the disease in urban environments, especially because the transmission cycles of *T. cruzi* are adapting to human dwellings and domestic species that may be their main food sources under this scenario [193].

These studies highlight a pronounced need for disease prevention, better monitoring of the management of environmental variables such as landscape disturbance, allowing for close monitoring of the impacts of its transformation on human health [1]. Prioritization of strategies for surveillance, control and involvement of the environmental sector to strengthen and carry out joint efforts are needed [194]. Changes need to be driven in socioeconomic and development policies around sustainable objectives to enact disease prevention and avoid an increase in *T. cruzi* transmission cycles in suitable habitats [193].

An increasing environmental challenge is the proximity of human settlements, both urban and rural, to natural habitats. This has led to multiple difficulties in the long-term control of wild populations of the vector, which frequently infest dwellings near fragmented habitats [167]. As a consequence, these conditions have especially favored the potential exchange between wild triatomines and mammals carrying *T. cruzi* [195], giving rise to sylvatic–domestic interfaces of the disease. This scenario not only highlights the relevance of ecosystem conservation, but also the need for timely host monitoring, vector control and prevention measures against possible interactions between sylvatic and domestic cycles, such as those that have been previously documented in a future context of changes in land use and climate change [193].

#### 2.6.2. Agricultural and Livestock Expansion

In particular, Chagas disease has been favored by agricultural and livestock expansion by fragmenting and transforming natural habitats. These events have allowed for the movement of vectors and their wild reservoirs, such as rodents and marsupials, into inhabited areas, increasing their contact with humans and domestic animals, and facilitating the transmission of *T. cruzi* [41]. This land use change also creates sylvatic–domestic interfaces, where triatomines can move between domestic and wild animals, enhancing the risk of infection. As in fragmented landscapes, where plants that are suitable refuges for disease vectors can be found, agricultural infrastructures, such as corrals and barns, play an important role as shelter and food to triatomines, promoting their establishment in inhabited areas and facilitating the colonization of dwellings by vectors and domestic transmission [61].

#### 2.6.3. Climate Change

Climate change has the potential to alter or expand the natural ranges of vectors, hosts, and pathogens and to convert some previously uninhabitable regions into suitable habitats [196,197,198,199]. Although triatomines prefer dry environments, moisture in certain locations can create favorable conditions for their refugia. Moist locations, such as areas near sources of standing water, can support the growth of plants and structures that provide shelter for these insects. Social and ecological factors interact together to influence the presence of the vector and the spread of Chagas disease. The integration of social and educational interventions with environmental control strategies is key to effectively addressing the disease.

#### 2.6.4. Factors Favoring the Presence of the Vector or the Disease

Social vulnerability influences the presence of the disease, showing a higher prevalence even in the low abundance of infected vectors [142]. Lack of basic services such as drinking water, sanitation and electricity can contribute to unsanitary conditions that favor the presence of triatomines. However, nowadays, with the invasion of wildlife habitats caused by accelerated urban growth, it is no longer considered a rural disease.

It is estimated that if urban growth continues, in the future, without adequate control of the disease, it may become a more acute public health problem in densely populated cities. Domestic mammals (dogs, cats) and species such as rats could play an important role as blood sources for triatomine bugs in the absence of wild mammals. The low level of awareness may lead to people not being aware of the necessary preventive measures or the importance of hygiene and housekeeping.

Cultural practices that favor close coexistence with domestic or wild animals, which may serve as a reservoir for triatomines, may increase the risk of infestation. Likewise, lack of cooperation with vector control programs due to cultural beliefs or distrust of health authorities can reduce the effectiveness of control measures. Migration and displacement of communities due to work, education, conflicts or natural disasters lead to the spread of the disease to areas where vectors were not present, thus facilitating the spread of the disease.

#### 2.6.5. Factors That Reduce the Presence of the Vector or the Disease

The intervention of public health programs involves access to education campaigns, where educational programs are provided to inform the population about Chagas disease, its vectors and preventive measures, increasing participation in control efforts. Housing improvement programs, such as rehabilitation and construction with appropriate materials, help to reduce the available shelters for the vector. Availability of access to health services for early diagnosis and treatment is critical. Improved sanitation and waste management, such as garbage disposal and reduction in shelters for triatomines, is one way to control the spread of the disease.

Fumigation and vector control programs, implemented regularly and effectively, can significantly reduce the triatomine population. These programs often require the active participation of communities to be effective. Government policies that support vector control, disease education and housing improvement can be very effective. Implementation and enforcement of these policies help create a more favorable environment for disease control. Proper surveillance and monitoring of vector presence and disease, in conjunction with research into new methods of control and prevention, are critical to disease and/or vector control.

### 2.7. Integral Recommendations for Chagas Disease Prevention

#### 2.7.1. Challenges of Wild and Domestic Triatomines in the Control of Chagas Disease

The control and eventual eradication of Chagas disease face significant challenges due to the complex interactions between wild and domestic triatomines. Wild triatomines remain a persistent obstacle as they exhibit high adaptability, enabling them to reinfest areas where domestic transmission has been previously reduced [195]. For instance, studies in the Gran Chaco region have shown that wild triatomines recolonize areas even after extensive vector control efforts, driven by their ability to exploit ecological disruptions such as deforestation and agricultural expansion [199]. This adaptability complicates control measures and underscores the need for continuous monitoring and innovative strategies. Their ecological behavior and wide distribution across diverse habitats—from forested ecosystems to peri-urban environments—pose a continuous risk for *T. cruzi* transmission.

Similarly, domestic triatomines present challenges linked to their close association with human dwellings and domestic animals, particularly in rural and peri-urban regions. Poor housing conditions, such as cracks in walls, thatched roofs, and inadequate sanitation, create ideal environments for their colonization and reproduction [67]. For instance, sealing cracks and crevices with cement, replacing thatched roofs with durable materials like metal sheets, and installing fine-mesh screens on windows and doors have been proven effective in minimizing vector entry and habitation. Additionally, ensuring proper sanitation and waste management in and around homes further disrupts triatomine breeding grounds. Additionally, the coexistence of domestic animals (e.g., dogs, chickens, and pigs) serves as a reservoir for *T. cruzi*, perpetuating transmission cycles within households [200].

#### 2.7.2. Challenges Demanding an Integrated and Differentiated Approach to Chagas Control

Wild triatomines: Specific control strategies must focus on detailed studies of the biology, ecology, and population dynamics of wild triatomines to understand dispersal patterns and reinfestation risks. The use of geospatial tools, such as GIS-based mapping, can help identify areas of greatest risk and design targeted interventions. Strategies may include environmental management, monitoring sylvatic populations, and mitigating land-use changes that increase human–vector contact.

Domestic triatomines: Control measures should prioritize housing improvement programs, including sealing entry points, replacing thatched roofs with solid materials, and applying insecticidal treatments where necessary. Community-based surveillance systems can play a key role in detecting and addressing infestations early. Moreover, efforts to manage domestic animal reservoirs through health checks, vaccination development, and exclusion from sleeping areas are essential.

#### 2.7.3. Effective Results and Responsible Organizations

Successful control of vector transmission has been demonstrated through sustained, region-specific interventions. For example, in Brazil’s northeastern region, the implementation of housing improvement programs combined with community surveillance has significantly reduced triatomine infestations [197]. These efforts, led by partnerships between local health departments and international organizations, provide a model of effective collaboration and highlight the importance of tailored strategies in endemic areas. For instance, community engagement programs in endemic areas have effectively reduced vector densities by over 70% in targeted regions. Similarly, housing improvement initiatives, such as those supported by international organizations like the PAHO and local health ministries, have significantly reduced domestic infestations. These efforts highlight the critical role of partnerships among government health agencies, academic institutions, and non-governmental organizations (NGOs) in achieving measurable outcomes. Strengthening collaboration and resource allocation among these stakeholders remains crucial to sustaining these achievements [201].

#### 2.7.4. Importance of Joint Community and Professional Preventive Strategies

Community-based preventive strategies are fundamental for the sustainable control of Chagas disease; however, they must be complemented by professional interventions to ensure long-term effectiveness. Effective integration can be achieved through initiatives that combine local knowledge with scientific expertise [202]. For example, while community members can be trained to identify and report triatomines, entomologists and health professionals can support these efforts by providing advanced diagnostic tools and conducting targeted insecticide applications. Additionally, joint workshops that bring together community leaders, health workers, and researchers can foster mutual understanding and the co-creation of tailored strategies that address both local practices and scientific insights. By aligning grassroots actions with professional interventions, these integrated approaches can enhance the sustainability and efficacy of Chagas prevention efforts. Home-based methods, such as the use of natural repellents and house cleaning, while valuable, cannot substitute systematic and scientifically based approaches. These include regular monitoring for the presence of triatomines, and the strengthening of entomological surveillance systems. To achieve comprehensive prevention, it is essential to implement joint strategies that bridge professional expertise with community empowerment. These strategies must consider the following key dimensions:

Cultural aspects: Tailor health education programs to local customs, beliefs, and languages to ensure that preventive measures resonate with the affected populations. For example, involving community leaders and respecting cultural practices around housing construction and maintenance will improve acceptance and adoption of interventions.

Scientific and technical solutions: Ensure that professional interventions, such as targeted insecticide applications and environmental management, are based on scientific evidence and adapted to local ecological conditions. Combining these with innovations such as geospatial tools for vector mapping and monitoring will enhance the precision and impact of control efforts.

Social empowerment: Promote active community participation through educational programs that emphasize the role of families and individuals in preventing infestations. Empowering local populations to identify and report triatomines, maintain clean and vector-proof homes, and manage domestic animals effectively creates a sense of ownership over disease control.

Political and governmental leadership: Advocate for strong political will and governmental commitment to support Chagas control programs. This includes securing consistent funding, establishing policies that prioritize housing improvements, and integrating Chagas disease prevention into national and regional health agendas. Additionally, fostering intersectoral collaboration among health, education, housing, and agricultural ministries will ensure coordinated and sustained interventions.

Economic considerations: Invest in infrastructure improvements, such as housing rehabilitation programs to eliminate cracks and replace vulnerable materials (e.g., thatched roofs), which serve as habitats for domestic triatomines. Allocating resources for community surveillance systems and local health workers can ensure regular monitoring and early detection of vector presence.

## 3. Conclusions

The control of Chagas disease remains a significant challenge due to the complex interplay of factors involving wild and domestic triatomines, socio-economic conditions, and ecological dynamics. For example, socio-economic conditions such as poverty, inadequate housing, and limited access to healthcare contribute to persistent domestic vector presence, while ecological factors like deforestation and urban expansion disrupt natural habitats, increasing human–vector contact. While substantial progress has been made in reducing transmission in many regions, the persistence of wild vectors, poor housing conditions, and inadequate health infrastructure continue to hinder eradication efforts. To effectively address these challenges, integrated approaches combining vector control, housing improvements, community education, and scientific innovation are essential. Sustained collaboration among governments, academic institutions, non-governmental organizations, and communities will be critical to achieving the long-term goal of Chagas disease elimination. By fostering intersectoral partnerships and promoting the development of novel tools and strategies, the global health community can ensure more effective and sustainable control efforts.

## Data Availability

Not applicable.
